# Mindfulness-Based Cognitive Therapy for Young People and Their Carers: a Mixed-Method Feasibility Study

**DOI:** 10.1007/s12671-017-0842-7

**Published:** 2017-10-27

**Authors:** Daniel N. Racey, Jerry Fox, Vashti L. Berry, Kelly V. Blockley, Rachel A. Longridge, Jennifer L. Simmons, Astrid Janssens, Willem Kuyken, Tamsin J. Ford

**Affiliations:** 1 Child Mental Health Group, University of Exeter Medical School, South Cloisters, St. Luke’s Campus, Heavitree Road, Exeter, EX1 1TE UK; 2 AccEPT Clinic, Mood Disorders Centre, University of Exeter, Sir Henry Wellcome Building, Streatham Drive, Exeter, EX4 4QG UK; 3 NIHR CLAHRC South West Peninsula (PenCLAHRC), South Cloisters, St. Luke’s Campus, Heavitree Road, Exeter, EX1 1TE UK; 4 Devon Child and Adolescent Mental Health Service, Evergreen House, Victoria Park Road, Exeter, EX2 4NU UK; 5 Oxford Mindfulness Centre, University of Oxford Department of Psychiatry, The Prince of Wales International Centre, Warneford Hospital, Oxford, OX3 7JX UK

**Keywords:** Mindfulness, Depression, MBCT, Parents, Young people

## Abstract

We aimed to evaluate whether mindfulness-based cognitive therapy (MBCT) was feasible and acceptable for young people, their parents and the clinicians working with them; whether a parallel course for parents was a useful addition; and whether attendance at MBCT was associated with improved outcomes. The design was a mixed-method service evaluation of an eight-session MBCT programme for young people who were recovering from depression. The course was a manualised eight-session group intervention. Both young people (*n* = 18) and parents (*n* = 21) completed validated measures before and after the course. Semi-structured interviews were completed with some group participants and clinical staff working in the service. Care records were searched for additional contact following the intervention. Qualitative data from young people, parents and clinicians suggested that MBCT was acceptable and feasible and provided strategies to cope. The parent course was reported to provide personal support to parents and helped them cope with their child’s depression whilst also impacting the family, promoted shared understanding of depression and strategies to combat it and addressed intergenerational aspects of depression. Eighty-four per cent of participants attended at least 6/8 sessions, and 48% required no further intervention within the following year. Young people had statistically significant improvements across all outcome measures, whilst parents had statistically significant improvements in rumination, self-compassion and decentring.

## Introduction

Depression is the leading cause of illness and disability worldwide among young people (Dick and Ferguson 2015). Although uncommon in younger children, the incidence of depression increases rapidly to a peak in the mid- to late teens (Kessler et al. 2001). There is strong continuity of psychiatric disorder from childhood into adolescence and adult life (Copeland et al. 2013; Ford et al. 2017; Kessler et al. 2012; Kim-Cohen et al. 2003). Those with a juvenile onset of depression experience greater impairments in social and occupational functioning and reduced quality of life compared with those whose first episode occurs in adulthood (Goodyer et al. 1997a, b; Kessler et al. 2001; Richardson et al. 2012). In addition, adolescence is a key life stage in which behaviours that may profoundly influence future health develop, such as diet, exercise, sexual activity and substance use, and when decisions related to education, occupation and child bearing can massively alter the young person’s trajectory (Sawyer et al. 2012). Poor mental health is intimately associated with poor physical health, and psychological distress predicts mortality from all causes (Russ et al. 2012), whilst impairing psychopathology in childhood is associated with exclusion from school, occupational failure, the breakdown of intimate relationships and criminality (Collishaw et al. 2004; Costello and Maughan 2015). The prevention of future episodes of depression together with the amelioration of residual subclinical symptoms among those treated in childhood should be a priority because of the many potential benefits that extend beyond the obvious need to alleviate current psychological distress.

Depression is an episodic condition; those who experience their first episode in childhood have approximately double the risk of their peers of succumbing to further episodes (Costello and Maughan 2015; Rohde et al. 2013). In adulthood, there appears to be a ‘kindling effect’: the risk of further depressive episodes increases with every consecutive episode (Ma and Teasdale 2004), whilst the onset of subsequent episodes are associated with fewer stressful life events (Kendler et al. 2000; Lewinsohn et al. 1998). In addition, parental depression predicts the persistence of depressive disorder in their children, as well as additional costs in terms of mental health-related service contacts (Costello and Maughan 2015; Ford et al. 2017; Knapp et al. 2015). Given these strong links between parental and child psychopathology, a simultaneous intervention for parents/carers (henceforth referred to as parents) and their children might be particularly powerful.

Although we have evidence-based treatments for acute depressive episodes in young people, we lack robustly evidenced relapse prevention interventions (Costello et al. 2002; Cox et al. 2012; Merry et al. 2004; Thapar et al. 2012). A recent Cochrane review retrieved only nine trials, which combined with the heterogeneity of methodology, allowed only limited synthesis. Antidepressant medication reduced the proportion of young people who experienced relapse to 40% from two thirds, whilst psychological approaches were encouraging but could only be studied in combination with medication (Cox et al. 2012). These findings indicate the need to develop and test additional therapeutic approaches for young people who partially respond and/or relapse after treatment for depression.

In adults, meta-analysis suggests that mindfulness-based cognitive therapy (MBCT) is an effective and cost-effective option for relapse prevention among those who have experienced three or more episodes (Kuyken et al. 2016). Mindfulness involves bringing a non-judgemental intention to attend to the present moment including bodily sensations, feelings, thoughts, and environmental stimuli (Kabat-Zinn 1994), whilst MBCT is an eight-session group intervention program that combines mindfulness practice with cognitive behavioural elements (Segal et al. 2002). Mindfulness training may increase the ability of people who have a tendency to get depressed to avoid dwelling on negative cognitions/ruminations, which is thought to play a role in the onset, relapse and maintenance of depression (Nolen-Hoeksema et al. 2008; Segal et al. 2002). Participants learn to recognise negative thinking and relate to it with greater self-compassion, enabling them to step back or decentre from it. MBCT appears to be as cost-effective as antidepressants in the prevention of depressive relapse in adults, which has led to its inclusion in statutory treatment guidelines (Kuyken et al. 2016).

Research into the use of mindfulness in young people is sparse (Weare 2013), but interest is growing rapidly (Tan 2016). As this evaluation focused on a targeted intervention for a clinical population, the promising trials of mindfulness based approaches as a universal preventive approach in schools falls outside our remit (Britton et al. 2014; Kuyken et al. 2013; Sibinga et al. 2013). While a small number of randomised control trials have applied mindfulness-based interventions in adolescent populations (Kallapiran et al. 2015), the interventions studied to date have not included parallel intervention for parents and have suffered from a number of methodological issues (Tan 2016), which include interventions delivered to young people with heterogeneous conditions and a failure to adequately describe the specifics of the mindfulness intervention applied. For example, Tan and Martin (2014) randomised 91 young people aged between 13 and 18 years from community mental health clinics to treatment as usual or a 5-week mindfulness programme. They demonstrated improvements in a number of mental health outcomes, although it is unclear which was primary, and whilst inclusion criteria stated psychiatric diagnoses, these were unspecified. Likewise, Biegel et al. (2009) tested young people aged 14–18 years with ‘heterogeneous diagnoses’ recruited from psychiatric outpatients with mindfulness-based stress reduction or treatment as usual and reported reduced anxiety, depression and somatic symptoms with improved sleep and self-esteem. Nearly half (49.0%) of the young people in this trial were reported to have a mood disorder, and a third (30.4%) anxiety, but comorbidity was common and nearly a quarter had other disorders (24.5%).

Although well-conducted randomised controlled trials provide the strongest type of evidence that an intervention is effective, they are expensive and time consuming, and therefore preliminary feasibility and piloting work is important to optimise the intervention and the research processes (Craig et al. 2008). We piloted a manualised MBCT intervention adapted for use with young people attending a Child and Adolescent Mental Health Service (CAMHS), who had completed first line treatment for depression and/or anxiety, with a parallel intervention for their parents. Specifically, we wanted to evaluate whether MBCT was feasible and acceptable for young people, their parents and the clinicians working with them, whether a parallel course for parents was a useful addition to the adult MBCT model and whether attendance at MBCT was associated with improved clinical outcomes. We anticipated that if effective, MBCT would be associated with a reduction in rumination and depressive symptoms and increased mindfulness, including self-compassion and decentring, thereby reducing the young people’s risk of relapse.

## Method

### Participants

**Fig. 1:**
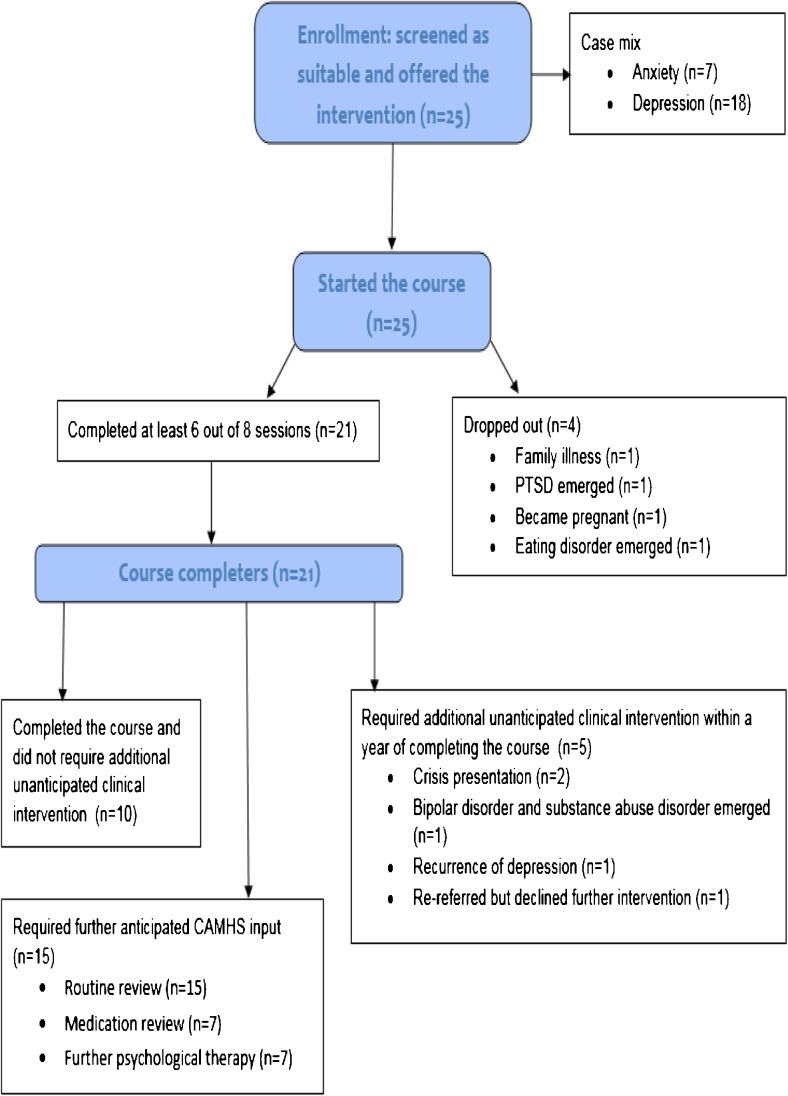
Flow of participants

Figure 1 demonstrates the flow of young people and carers through the intervention; young people (aged 14–18 years) were recruited within a single CAMHS in Devon. They were referred by CBT-trained care coordinators as having partially recovered from the acute episode of depression, with or without a comorbid generalised/phobic anxiety disorder. Exclusion criteria were young people who presented with a high level of risk to self or others or were the subject of safeguarding concerns; young people or parents who were experiencing an acute episode of depression, psychosis, eating disorder, obsessional compulsive disorder or post-traumatic stress disorder (PTSD); had active substance misuse difficulties; attention deficit hyperactivity disorder, conduct disorder; or who were actively engaged in other forms of psychological therapy.

### Procedure

This study was conducted as a service evaluation that was registered with the CAMHS audit and quality improvement team in the service from which these data were collected. All participants provided written informed consent and were aware that an intervention used with adults was being adapted, and its use with young people and their parents was being evaluated. They were aware that their pseudonymised data would be analysed for presentation and publication.

Parents and young people were provided with written information about the service evaluation prior to their orientation meeting with the group leaders to discuss the course. Written consent was collected after the course, and the evaluation had been discussed with the parents and young people in person. We present data for young people and parents who attended four separate MBCT groups, which were run between 2014 and 2016. Participants completed the measures described below at the beginning of the first and last sessions. If a young person or their parent dropped out, the remaining member of the dyad was encouraged to continue attending the group, and both parents could attend if they wished to.

The intervention was adapted for young people and their parents using the existing MBCT manual for adults (Segal et al. 2002) and comprised eight sessions with separate groups for young people and for parents, run in parallel. The programme was designed to enable young people to become more aware of the bodily sensations, thoughts and feelings associated with depression and to learn to relate to these more constructively. The first four sessions of the young person’s group aimed primarily to increase their awareness of inattention and increase their ability to direct their attention to the experience of the present moment. Sessions involved a series of explanations, focused exercises and group meditation practices, followed by small and large group discussions. Participants were encouraged to reinforce this with formal practice at home between sessions. Sessions five to eight aimed to increase young people’s awareness of habitual patterns of reactivity and associated judgements and behaviours, as well as the development of new ways of relating to thoughts and feelings. As with the first half of the course, it combined instruction, exercises, meditation practice and discussion in the session, with individual practice at home. The parent’s group followed a similar programme that additionally aimed to provide parents with the understanding and the skills to enable them to recognise their child’s and their own negative emotional states, and to empower them to step out of their own automatic/habitual patterns of thinking and behaving, as well as to assist their child to do likewise.

At the final session, participants in groups one and three were invited to undertake a semi-structured interview to gather data about their experience of the course and how it might be improved. Additional information sheets and consent forms were provided to those who expressed an interest. These interviews were conducted by RL, who was independent of group delivery, at a time and place of the participant’s convenience. The topic guide for young people and parents covered whether anything had changed for them that they attributed to the course and perceived mechanisms of change, systemic impacts on relationships and the wider family, their awareness of mindfulness and expectations prior to the course and their experience of the course. The interviewer asked specifically about the parallel sessions for parents and aspects of the course that they found difficult.

In the spring of 2016, all clinical staff (*n* ~ 60) who worked within the CAMHS where these courses were delivered were e-mailed with an invitation to participate in brief semi-structured interviews about their expectations, beliefs and concerns about the intervention, as well as their level of understanding about the evidence base of MBCT. One person responded directly, whilst a purposive sample of staff from different teams, initial training and levels of experience of both child mental health and mindfulness was selected and approached individually. Consent was obtained and brief interviews were conducted with all but three members of staff approached during the spring of 2016. The characteristics of the achieved sample are reported in the results. The aim of these interviews, conducted by JS, was to explore the acceptability and possible position of MBCT within the wider CAMHS context (National Collaborating Centre for Methods and Tools 2011). The topic guide for clinicians covered their understanding of MBCT, its potential as an intervention for their patients, the utility of a parallel intervention for parents and factors that might influence their making a referral to future groups.

Care records were searched (RL/JS/DR) for reports of additional unpredicted contact with CAMHS among the young people who attended the group during the year following their MBCT course. Where young people left the service (moved area or turned 18), we made contact with them or their parents to discover if they had needed additional care.

### Measures

All measures were completed by both young people and their parents. They were selected to allow comparison between our work and others studying mindfulness and depression, and all were reviewed by young people for appropriateness to their age group and context.

#### Beck Depression Inventory-II (Beck et al. 1996)

The Beck Depression Inventory-II (BDI-II) is a 21-item self-report instrument developed to measure severity of depression with an emphasis on affective and cognitive symptoms, which is sensitive to change, including in MBCT trials (Williams et al. 2008). Each item is rated on a 4-point scale from 0 to 3 and a total score is created by summing all 21 items. Higher scores represent greater severity of depression. The following symptom severity ranges have been specified: minimal (0–13), mild (14–19), moderate (20–28) and severe (29–63). The BDI-II has excellent internal consistency with an alpha coefficient of .94 (Arnau et al. 2001) and good convergent validity (Arnau et al. 2001). In young people aged 13 years or more, scores correlate highly with the Major Depression subscale of the Diagnostic Interview Schedule for Children (Wilcox et al. 1998).

#### Rumination Response Scale (Treynor et al. 2003)

The Rumination Response Scale (RRS) is a 22-item self-report instrument developed to measure rumination, a method for coping with negative mood involving self-focused attention. Each item is rated on a 4-point scale from 1 = almost never to 4 = almost always. A total score is created by summing all 22 items. Higher scores represent a greater level of rumination. The RRS has an internal consistency of .90 and test-retest correlation of .67 (Treynor et al. 2003). Alterations and adaptations to the RSQ scale have been made in the past for studies with children (Children Response Styles Scale (Ziegert and Kistner 2002); Children’s Response Styles Questionnaire (Abela et al. 2002)), but there was no indication of whether the rumination items from these adapted measures could be extracted and applied independently, so we used the adult version.

#### Self-Compassion Scale (Neff 2015)

Beneficial changes in self-compassion are an intended outcome of MBCT. There is a long and short (Self-Comparison Scale (SCS)-SF) version of the SCS but correlation between the two is very high (.98). We used the long (26 items) version for this paper to measure self-compassion in both young people and adults. The self-compassion scale alpha is .93 for total score for samples of undergraduate students (mean age 19) (Neff 2015). The questionnaire was reviewed by a young person, who considered that the questions were easy to understand and rate. Responses are scored on a 5-point scale from 1 = never or very rarely true to 5 = very often or always true, with items 1, 4, 8, 9, 11 and 12 reversed scored. Higher scores indicate higher levels of self-compassion. A total score is created by taking a mean of all items.

#### Mindful Attention Awareness Scale (Brown and Ryan 2003)

This 15-item self-report instrument was used to measure changes in mindfulness in parents and young people, with higher scores indicating a higher level of mindfulness. Respondents are asked to rate a series of statements on a six point scale anchored between ‘almost always’ (1) and ‘almost never’ (6). A total score is created by taking a mean of all 15 items. Exploratory and confirmatory factor analysis suggested that the Mindful Attention Awareness Scale (MAAS) assesses a single construct, and the same series of studies in adults suggested that the measure has good test-retest reliability (intraclass correlation .81), internal consistency of .85 and high convergent and divergent validity (Brown and Ryan 2003). The adolescent version (MAAS-A) contains 14 items (Brown et al. 2011), which are identical to the adult measure (that contains one additional item), hence our decision to use the latter. Cronbach’s alpha is .80 in both healthy and psychiatric samples.

#### The Experiences Questionnaire Decentring Subscale (Fresco et al. 2007)

The Experiences Questionnaire is a 20-item instrument developed to measure both rumination and decentring, which is the ability to take a detached view of one’s thoughts and emotions. The decentring subscale is an 11-item measure where respondents are asked to indicate how often they are able to achieve statements of decentring, such as ‘I can observe unpleasant feelings and not be drawn into them’, on a 5-point Likert scale ranging from 1 = almost never to 5 = almost always. A total score is created by summing all 11 items. Higher scores represent greater ability to be decentred. This measure is not yet formally validated for young people but the decentring subscale had a Cronbach’s alpha of .83 among undergraduate students (mean age 19 years). It was selected, after review by a young person, as it had face validity about a key mechanism that we wished to explore, diminished emotional reactivity.

### Data Analyses

All semi-structured interviews were supported by a topic guide and were audiotaped and transcribed. The interviews were analysed using NVivo 11 to conduct a thematic analysis and both inductive and deductive themes were extracted from the data. The topic guide was developed by DR and RL with a mixture of a priori hypotheses and inductive themes that emerged from initial interview scripts after repeated review to highlight themes. The themes and the topic guide were then reviewed by AJ who provided critical feedback and identified additional themes, which allowed us to refine the topic guide for the second group that were interviewed and informed our subsequent thematic analysis. Similarly, the topic guide for clinicians was developed by JS and DR using the same method, combined with review by VB and TF.

Quantitative analysis was conducted using SPSS version 22. We assessed feasibility by attendance at the course and retention. In order to assess whether the intervention may have produced changes related to mindfulness, we compared changes in self-compassion, mindfulness and decentring scores before and after the intervention using paired *t* tests. Given the small numbers attending each group, data analysis for all four groups was combined (group A, eight young people and nine parents; group B, five young people and five parents; group C, six young people and eight parents; group D, seven young people and eight parents—one young person and one parent from this group did not complete the research measures).

## Results

After a description of the samples achieved, we report the qualitative and quantitative results in relation to feasibility of MBCT in general, the utility of the parent group and clinical outcomes.

Baseline data on clinical outcomes were collected from between five and eight young people at each of the four groups (total = 25) and between five and nine parents (total = 29); sometimes both parents wished to attend. Follow-up data on the self-report BDI were available for 21 parents and 18 young people. Both young people and parents were predominantly female, whilst reports of past and family history of depression and current antidepressant use were extremely common from both informants (see Table 1).

**Table 1:** The characteristics of parents and young people who participated in the evaluation

Characteristic	*N*	Value
Young people (*n* = 25)		
Mean age (years (*R*, SD))^a,b^	22 (88%)	16.4 (14–18, 1.0)
Girls (*n*)	25 (100%)	23
History of depression (*n*)	21 (84%)	18
Taking medication for mood (*n*)	21 (84%)	9
Mean number of episodes (*R*, SD)	13 (52%)	1.9 (1–7, 1.7)
Mean number of relatives with depression (*R*, SD)	18 (72%)	1.6 (0–4, 1.0)
Parents (*n* = 29)		
Mean age (years (*R*, SD))	29 (100%)	47.8 (36–53, 5.0)
Women (*n*)	29 (100%)	28
History of depression (*n*)	29 (100%)	16
Taking medication for mood (*n*)	28 (97%)	8
Mean number of episodes (*R*, SD)	16 (55%)	1.3 (0–5, 1.5)
Mean number of relatives with depression (*R*, SD)	28 (97%)	2.0 (0–5, 1.3)

*N* number providing data, which varies as not all participants completed every measure^a^ Range^b^ Standard Deviation

A total of nine parents (two fathers) and seven young people were interviewed; two young person-parent dyads chose to be interviewed separately, four were interviewed together and one young person was interviewed with both parents. One parent was interviewed alone as their child chose not to participate.

Twenty-one interviews were completed with practitioners from a range of professional and/or therapy backgrounds, nine of whom had referred young people into the MBCT group. A few clinicians in the service from which these young people were recruited had completed an 8-week basic course. Only one was a trained mindfulness practitioner so outside the groups, exposure to mindfulness for the participants would have been very limited and would have consisted of breathing and grounding techniques in a very few cases. The sample comprised eight nurses, three primary mental health workers, two clinical psychologists, two counsellors, and one of each of the following: social worker, occupational therapist, health care assistant, psychiatrist, drama therapist and cognitive behavioural therapist. One of those interviewed was a trained mindfulness practitioner, whilst the others reported a range of experience of mindfulness that extended from six with ‘a lot’, five ‘moderate’ five ‘a little’ experience and four with ‘none’. These clinicians had a variety of clinical roles, which included service manager (1), anxiety and depression pathway (9), self-harm team (3), primary mental health pathway (3), under 12 s (2), neurodevelopmental difficulties team (2) and the joint agency child abuse team (1).

### Acceptability and Feasibility

As Fig. 1 illustrates, 21 of the 25 young people and their parents who started the course attended at least six or more of the eight sessions, which suggests that the intervention was feasible and acceptable to most of those deemed eligible. Three of the four young people who attended fewer sessions presented with previously undetected psychiatric comorbidity or relapse, which would have rendered them ineligible had these difficulties emerged earlier.

Overall, the interviews with young people and parents suggested that both parents and young people found participation to be a positive experience that taught them skills that they planned to continue to use. As illustrated in Table 2, a strong theme was that young people and parents found the intervention strange initially.

**Table 2:** Summary of the qualitative evaluation regarding acceptability and feasibility with parents, young people and clinicians

Theme (respondents)	Supporting quote
Initial response to the intervention; seemed a bit strange (all informants)	YP06 ‘I think what a lot of people imagine when they hear like mindfulness and meditation is they imagine like Tibetan style flags and joss sticks and ommmm’. P11 ‘…I did think for the first couple of sessions… this is a bit odd, this is a bit strange, I am not too sure. But beyond that, you can… understand the process…’ PneE16 ‘too alternative for some’
What is MBCT? (clinicians)	U12nE10 ‘I’m not sure how it interfaces with cognitive behavioural therapy...’
Who is MBCT for? (clinicians)	AdnE14 ‘I think that’s to do with not being clear about when mindfulness is appropriate or not. So therefore it’s more difficult to refer. Because there are a lot of assumptions that everybody understands mindfulness’. ShE15 ‘I think the keys factors are that 1. The young person is stable in terms of their mental health, in other words that their risk has reduced. That they are around about halfway to three-quarters away to the end of their CAMHS treatment journey, so that there is some preparation in the keyworker or key therapist’s plan that they are moving towards discharge’. AdnE017 ‘I think they need to be in a place where [...] they have had already and have an understanding of CBT concepts, so that they have a sense of, you know the processes to do with your thoughts and feelings and behaviour and what you do, because that all comes into the programme, as far as I understand, and that would make them better prepared’.
Who might MBCT not work for? (clinicians)	AdE07 ‘We decided that it would be too exposing in the group if everybody else was doing the body-breath stuff and she could not do that, it probably would not be helpful at this time. So I am working with her individually’ NenE12 ‘young people within the neurodevelopmental pathway might be a little more difficult to apply that typical sort of therapy’.
Time commitment (all informants)	P11 ‘It’s a busy lifestyle and you have to snatch it where you can...when you have got a busy lifestyle and family, taking time out for 20, 25 min is quite difficult’. P12 ‘I was going to try to listen to the podcasts morning and evening, regular and organised and it has not really worked out like that’. P08 ‘It’s not like you must practice for half an hour every day...It’s what fits into your life’.

The speciality and experience level of the practitioners are reflected in the participant identification codes used for the qualitative data and correspond to the below:

Ad, anxiety and depression; Sh, self-harm; Ne, neuro; U12, under 12 s; Pm, primary mental health team; E, experienced (previously run mindfulness groups, taken courses or conducted research into mindfulness); nE, not experienced (may know in principle what mindfulness is about but do not regularly use for work or personal reasons); P, parent; YP, young person

Mindfulness interventions often stress the difference between formal and informal practice. Formal practice is dedicated time which has been set aside from interruptions whilst informal practice is more spontaneous and happens in reaction to everyday contexts. Our course, in common with others, expects participants to formally practise regularly, but almost universally young people and parents reported that they were too busy, although they used informal practice in everyday life and found this useful.

Many of the clinicians interviewed reported that they were confused by the distinction between ‘MBCT’ and ‘mindfulness’. Some clinicians expressed concerns that the time commitment for parents and young people might prove overwhelming, as might participation in a group situation, and that the intervention might be too ‘alternative’ for some of their clients (PneE16). Some clinicians were worried by the lack of evidence-based whilst others raised practical issues such as confusion about referral processes, the distinction from other modes of therapy or the place of MBCT within care pathways. However, the option to refer to MBCT was seen by most practitioners as a positive additional intervention, specifically for young people with more long-term effects of recurrent depression and for those with rumination. Practitioners were aware that MBCT might not be a suitable approach for young people with PTSD, as well as those with a high level of risk or in the acute phase of their illness. Another theme raised by clinicians was the need for cognitive understanding about thought processes, which was mentioned both in the context of neurodevelopmental disorders, young people who had no prior experience of CBT, younger children and those with learning disability.

### The Utility of a Parallel Course for Parents

As Table 1 illustrates, more than half of the parents had a personal history of depression, more than a quarter were currently taking antidepressant medication and many also reported a strong family history. As Table 3 illustrates, parents reported that the course was personally supportive and increased their capacity as a parent with a beneficial impact on the wider family. The impact on the family was also endorsed by young people; both young people and their parents found the shared experience of attending together mutually supportive and in some cases this helped to rebuild previously damaged relationships. Parents reported that their improved understanding into the processes that maintain depression increased both their empathy towards young people and provided an explanatory model for an illness that some found difficult to understand. Parents expressed how difficult they had found their child’s mental illness to cope with, and that sharing this with others was extremely helpful.

**Table 3:** Summary of the qualitative evaluation regarding the utility of the parent group with parents, young people and clinicians

Theme (respondents)	Supporting quote
Personal support for parents (all informants)	P12 ‘If you can manage yourself, you are in a better place to help them...If the home is a calmer and more supportive place. It might make life easier for everybody’. YP03 ‘I am glad that mum did the course at the same time as me doing it…she’s under kind of an external pressure and she’s starting to get upset’. U12E03 ‘when you have got young people that are highly agitated, or really struggling cognitively that their parent are also highly anxious as well. So it helps parents to work alongside them to help support them within the family’. PmnE19 ‘[Parallel groups] would be a very useful thing for an anxious parent, or a parent that tends to overthink’.
Impact on family (all informants)	P06a ‘All of us feel it if she’s under an external pressure and she’s starting to get upset so I think that’s really helped and that’s helped everyone else be a bit calmer as well’. YP12 ‘I think everyone feels the calm a bit more… I think it makes it easier on all of us to be honest’. YP03 ‘I am glad that mum did the course at the same time as me doing it… that’s helped everyone else be a bit calmer as well’.
Shared understanding (all informants)	P11 ‘When your child is not well and your life’s changed so much, it’s something that’s bringing you back together, even if it is something small’. P12 ‘Although I wanted to be understanding and supportive before...sometimes I would think oh, come on, surely you can find something that’s good in your life, it’s not really that bad. They would say something and think really is that what’s going on inside? I had no idea that was going on’. P11 ‘It’s helped me understand my daughter as well and what she must be going through. Unless you have experienced it [the course], I do not think you can support your child with it’. YP06 ‘one of the things with depression especially is how incredibly isolated you can feel at times… And that everything you do is a failure, and that it’s only [you] that’s feeling like that. … coming with adolescents alone makes you feel more like part of a group. But when you come home and turn off the lights and go to bed, you still feel alone. Alone in your experiences, alone in what you have done. …. But if you have one or two other people within the household who have done the same thing, you feel like you are not alone. It’s also really helpful to know that there are other people who are doing it. And it’s really helpful to know that you are all in the same boat. Other families, other adolescents, adults, household members’.
Coping with the impact of depression on parents (parents and clinicians)	P07 ‘We’d both had problems. Mine were a lot about (YP). It was so stressful living with her...it was so difficult’. P12 ‘As a parent, you feel as if you have failed somehow...You want your children to be happy so if they say they are not then you feel deeply sad’.
Potential to influence the intergenerational aspects of depression (clinicians)	AdnE21 ‘usually our young people who need these techniques their parents are suffering as well, and if they learn techniques it’s particularly useful that they can support their child’. ShE02 ‘So there’s two things for me—one is how the child’s parents are part of the solution and can help the child, and the other is how the parent also learns how to support and manage their own emotions, behaviours and thoughts’.

The speciality and experience level of the practitioners are reflected in the participant identification codes used for the qualitative data and correspond to the below:

Ad, anxiety and depression; Sh, self-harm; Ne, neuro; U12, under 12 s; Pm, primary mental health team; E, experienced (previously run mindfulness groups, taken courses or conducted research into mindfulness); nE, not experienced (may know in principle what mindfulness is about but do not regularly use for work or personal reasons); P, parent; YP, young person

Practitioners also considered the parallel group with parents to be a positive option with three key themes emerging: the potential to positively influence the intergenerational aspects of mental health, the benefits of shared understanding and practise, and the ability of the joint intervention to target the home environment. They also raised a few concerns about the provision of a course for parents, which revolved around the practical issues of getting parents and children to come to an intervention, and what would happen when one or other party could not, or did not want to, attend.

### Clinical Outcomes

The young people and parents interviewed understood mindfulness as increased awareness of self through thoughts, emotions and sensations and an ability to decentre from these experiences. They also believed that it was a generalisable skill (Table 4).

**Table 4:** Summary of the qualitative evaluation regarding clinical outcomes with parents, young people and clinicians

Theme (respondents)	Supporting quote
Strategies to cope (all informants)	YP08 ‘Mindfulness is maybe appreciating what’s actually worth worrying about and what’s not’. YP02 ‘…anyone could use it whether they were depressed or had any kind of mental issues at all could kind of use it to help in everyday life to reduce like nerves or spasms or stress or worries… It’s a life skill that I can carry on using’. U12nE10 ‘[MBCT] may be a useful adjunct, or a way of kind of consolidating the work that they have been doing so that that can be a kind of addition to the work they have doing within the service’.

The speciality and experience level of the practitioners are reflected in the participant identification codes used for the qualitative data and correspond to the below:

Ad, anxiety and depression; Sh, self-harm; Ne, neuro; U12, under 12 s; Pm, primary mental health team; E, experienced (previously run mindfulness groups, taken courses or conducted research into mindfulness); nE, not experienced (may know in principle what mindfulness is about but do not regularly use for work or personal reasons); P, parent; YP, young person

As Table 5 demonstrates, the mean BDI score for young people was within the moderate depression range at baseline, so despite having accessed prior treatment, these young people were not recovered. There were statistically significant improvements for young people who returned data across all of the outcome measures, in particular the BDI (from a mean of 21.1 at the start of the course to 12.4 after the final session (*p* = .004; CI, − 14.1 to − 3.2)). Parents also reported statistically significant reductions in rumination and statistically significant improvements in decentring and self-compassion. While not statistically significant, the changes in parental depression scores (*p* = .139; CI, − 4.4 to .7) and mindfulness (*p* = .162; CI, − .4 to 1.9) were in the anticipated direction.

**Table 5:** Young people’s and parents’ scores on quantitative outcome measure pre- and post-attending the MCBT course

Measure	*N*	Before (B) mean (SD)	After (A) mean (SD)	Mean change (A–B) (95% Confidence interval)	*p* value
YP Beck Depression Inventory	18	21.1 (11.3)	12.4 (12.1)	− 8.7 (− 14.1 to − 3.2)	.004
YP Rumination Response Scale	18	57.0 (15.5)	45.7 (15.3)	− 11.3 (− 17.9 to – 4.7)	.002
YP Self-Compassion Scale	18	2.5 (0.7)	3.0 (0.8)	0.5 (0.08 to 0.9)	.022
YP Mindful Attention Awareness	7	3.2 (1.0)	4.1 (0.6)	0.9 (0.1 to 1.7)	.040
YP Decentring	14	30.6 (7.5)	37.2 (7.3)	6.6 (2.9 to 10.3)	.002
P Beck Depression Inventory	21	8.6 (7.7)	6.7 (8.4)	− 1.9 (− 4.4 to 0.7)	.139
P Rumination Response Scale	20	39.3 (15.5)	32.2 (9.8)	− 7.1 (− 14.1 to – 0.03)	.049
P Self-Compassion Scale	21	3.0 (0.9)	3.4 (0.9)	0.3 (0.06 to 0.6)	.020
P Mindful Attention Awareness	8	3.9 (1.2)	4.7 (0.8)	0.8 (− 0.4 to 1.9)	.162
P Decentring	17	31.3 (8.5)	37.9 (6.5)	6.6 (3.2 to 10.0)	.001

YP, young person; P, parent, *N*, number with complete data at both time points; SD, standard deviation

The case records accessed showed that 10 of the 25 young people who started the course were discharged with no further contact over the following year (Fig. 1). Service contacts among the remaining 15 mainly comprised planned routine reviews (*n* = 15), some of which were to check maintenance medication (*n* = 7), further psychological therapy (*n* = 7) and crisis presentations (*n* = 2). Four young people transitioned to adult services, which suggest significant ongoing mental health need.

## Discussion

This small, uncontrolled evaluation provides tentative evidence that MBCT may have a role in the promotion and maintenance of recovery among young people who have experienced depressive disorder. Reports from all three key stakeholders were mostly positive. Practitioners thought that MBCT was a useful addition, particularly for those with more long-term affective difficulties, whilst the high levels of engagement, retention and positive experience reported by young people and parents suggest that MBCT was acceptable for these participants. The baseline depression scores, levels of medication and strong family and past history of depression indicate that we recruited a vulnerable group of young people and parents in terms of the risk of relapse. Most of those who did not complete the course developed comorbid psychiatric disorders that challenged their ability to access it and would have rendered them ineligible if detected at the screening assessment. This highlights the need for careful screening and ongoing monitoring of young people entering MBCT programmes.

It is worth noting that although initially conceptualised as a relapse prevention intervention, the baseline scores of the young people providing quantitative data suggest that they were still moderately depressed. This suggests that practitioners were referring young people for MBCT who may have limited therapeutic options in current CAMHS provision, other than antidepressant medication, which 43% were already taking. Given these high levels of medication prescription, future studies need to consider the role of antidepressants. Might MBCT allow lower doses to be used, or for medication to be taken for a shorter duration? The multimodal study of ADHD suggests that effective psychotherapy can reduce the dose of medication required and it may be that MBCT might reduce the duration and/or dose of antidepressant medication as it seems to in adults (Kuyken et al. 2015; Swanson et al. 2017).

There were some useful prompts about how to improve the acceptability of MBCT. Parents and young people reported thinking that the intervention was strange initially, which echoed clinicians’ concerns that it might be too ‘alternative’ for some families and suggests that careful preparation about what it entails may improve retention and engagement. However, it is likely that the approach will always seem unusual, and perhaps appear too ‘odd’ for some families to participate regardless of the support and explanation provided. Both parents and young people reported difficulty in finding time to practise formally, despite finding the skills learnt useful to deploy informally, which suggests a need to make formal practice more palatable and easier to schedule, and/or explore the utility of informal practice in terms of dose response. Practitioners reported being uncertain about what MBCT was in relation to mindfulness, how it should fit within current service organisational pathways, and raised concerns about groups for whom it may not be an appropriate option in its current form or indeed ever. This would suggest an appropriate level of caution among practitioners about the introduction of a novel therapy and the need for clear communication about referral criteria, as well as the need for better evidence of effectiveness in terms of routine outcome monitoring and more formal trials.

The parallel course for parents was enthusiastically endorsed by all groups of informants, which suggests that it is an acceptable intervention for CAMHS to provide. The intergenerational aspects of depression are well described (Hammen et al. 2012), and Wilkinson et al. (2009) demonstrated that severity of depression among young people was positively correlated with both maternal and paternal psychopathology across time. The high levels of personal and family history of depression indicate that these parents were also highly vulnerable to future depressive episodes. The parallel changes detected in depressive symptoms, self-compassion and mindfulness scores among parents suggest that an intervention that develops mindfulness skills among parents as well as the vulnerable young people that they care for may be particularly powerful and cost-effective, as their attendance may amplify the impact of MBCT on young people’s mental health whilst bolstering that of their parent(s). Reduced emotional reactivity within the family may be a mechanism whereby MBCT influences the mental health of both young people and their parents, which should be tested in future studies. Our qualitative data indicate some other potential mechanisms that future studies could explore to demonstrate whether this is the case. Parental reports indicated just how distressing it is to have a child with significant mental ill-health and how much they valued the peer support as well as gaining a better understanding of their child’s depression. Both parents and young people reported improved relationships within the wider family, shared understanding of depression and its impact as well as positive changes in the way the family functioned. Importantly, practitioners’ concerns that the commitment would be too great were not born out in this small sample, although it requires further testing and sensitivity is required for when one or other party is not able or willing to attend.

In terms of clinical outcomes, parents and young people reported that MBCT provided them with generalisable skills that helped them cope with stresses, which translated into statistically significant changes in all of the outcomes measured for children and three out of five of them for parents. The follow up of the Adolescent Depression Antidepressants and Psychotherapy (ADAPT) trial suggested that exposure to adverse life events predicted a diagnosis of depression at 28 weeks, which suggests that an intervention that improved resilience would have an important role in maintaining recovery (Wilkinson et al. 2009). For both young people and parents, symptoms of depression and the rumination thought to maintain/precipitate it reduced at the end of the course, whilst self-compassion, decentring and mindfulness scores increased, which suggests that MBCT may increase these skills as theoretically predicted. Importantly, the measures chosen to assess all of these constructs appear sufficiently sensitive to change, so could be usefully employed in future work. Nearly half (48%) of those who attended six or more sessions required no additional contact with CAMHS in the subsequent year, but there is a lack of recent data on the longitudinal trajectory of young people who have received treatment for depression in CAMHS to compare this with.

The predominance of girls and mothers reflects presentations to CAMHS with depression in the UK, and the epidemiology of depression in young people. That mothers were more likely to attend the group than fathers may be a combination of role allocation, availability (although the groups were in the early evening to avoid school/college) and personal history of depression. These would be interesting factors to explore in a process evaluation in a future trial of the intervention.

This evaluation addressed an important unanswered clinical problem; how to complete and maintain recovery among young people who have had depression and shows tentative evidence with quantitative measures that should inform the design of further research. It adapted an intervention with an established track record in adults for the adolescent population by involving parents and established the acceptability and feasibility of MBCT both for service users and the clinical setting in which it is likely to be delivered as well as providing important information to help further develop the intervention and its implementation. The study underlines the transgenerational nature of depression and points to mechanisms of change that can be studied further. To our knowledge, only one other study (Ames et al. 2014) has narrowed the focus of the intervention to depression among young people, with similarly encouraging findings.

### Limitations

Without controls, concealment and randomisation however, feasibility studies are subject to a number of measurement biases and should not be taken as evidence of effectiveness. We lacked detailed information about the background of participants as we were highly constrained in what data were acceptable and feasible to collect in an unfunded service evaluation. Similarly, participants completed measures with little support, so that missing data, particularly the Mindfulness Attention and Awareness Scale, limited power. Ideally, we would have included a standardised diagnostic assessment, along with measures of anxiety symptoms and formal practice, which would be important constructs to measure in future work. We chose measures that would allow comparison with the mindfulness literature, but not all of the measures have been validated for and/or used with young people. As research on mindfulness in young people is extended, parallel work is required to ensure a suite of measures that are demonstrated to have robust psychometric properties in this population. The inevitable imprecision that attends small samples may combine with publication bias to overestimate the effectiveness of novel interventions (Kraemer et al. 2006). Our findings can merely indicate an intervention with promise that should be tested formally; the changes detected on the questionnaires will be invaluable in calculating the sample size required to demonstrate effectiveness in a future trial, whilst the qualitative data offer important prompts to optimise the intervention as well as additional areas to explore quantitatively in subsequent work.

In conclusion, this MBCT intervention for young people who have completed first-line interventions for depression and who are not yet ready for discharge shows promise and should be tested further. The addition of a parallel group for parents was both feasible and acceptable and may amplify the benefits of the intervention for others in the family, as well as the identified patient.
